# A stoic and altruistic orientation towards their work: a qualitative study of healthcare professionals’ experiences of awaiting a COVID-19 test result

**DOI:** 10.1186/s12913-020-05904-0

**Published:** 2020-11-11

**Authors:** Malene Missel, Camilla Bernild, Ilkay Dagyaran, Signe Westh Christensen, Selina Kikkenborg Berg

**Affiliations:** 1 Clinical nurse specialist at the Department of Cardiothoracic Surgery, Centre for Cardiac, Vascular, Pulmonary and Infectious Diseases, Rigshospitalet, Copenhagen University Hospital, Copenhagen, Denmark; 2 Centre for Cardiac, Vascular, Pulmonary and Infectious Diseases, Rigshospitalet, Copenhagen University Hospital, Copenhagen, Denmark; 3 Clinical nurse specialist at Department of Infectious Diseases, Centre for Cardiac, Vascular, Pulmonary and Infectious Diseases, Rigshospitalet, Copenhagen University Hospital, Copenhagen, Denmark; 4 National Institute of Public Health, University of Southern Denmark, Odense, Denmark; 5 Department of Clinical Medicine, Faculty of Health and Medical Sciences, University of Copenhagen, Copenhagen, Denmark

**Keywords:** 2019-nCoV, COVID-19, Coronavirus, Qualitative study, Testing, Healthcare professionals

## Abstract

**Background:**

Extensive measures to reduce person-to-person transmission of COVID-19 are required to control the current outbreak. Special attention is directed at healthcare professionals as reducing the risk of infection in healthcare is essential. The purpose of this study was to explore healthcare professionals’ experiences of awaiting a test result for a potential COVID-19 infection.

**Methods:**

Qualitative interviews with 15 healthcare professionals were performed, underpinned by a phenomenological hermeneutical analytical framework.

**Results:**

The participating healthcare professionals’ experiences of awaiting a COVID-19 test result were found to be associated with a stoic and altruistic orientation towards their work. These healthcare professionals presented a strong professional identity overriding most concerns about their own health. The result of the coronavirus test was a decisive parameter for whether healthcare professionals could return to work. The healthcare professionals were aware that their family and friends were having a hard time knowing that the COVID-19 infection risk was part of their jobs. This concern did not, however, cause the healthcare professionals to falter in their belief that they were doing the right thing by focusing on their core area. The threat to own health ran through the minds of the healthcare professionals occasionally, which makes access to testing particularly important.

**Conclusion:**

The participating healthcare professionals had a strong professional identity. However, a discrepancy between an altruistic role as a healthcare professional and the expectations that come from the community was illuminated. A mental health coronavirus hotline for healthcare professionals is suggested.

## Background

The COVID-19 pandemic puts healthcare professionals (HCP) under pressure both physical and psychological [1]. The challenges include increased workload created by the outbreak but also fears of contagion for themselves, their families and patients. Particularly psychological health outcomes and distress are highlighted in current research regarding the initial stage of the COVID-19 outbreak in terms of anxiety, depression and post-traumatic symptoms [1–5]. Across these studies HCPs working during the epidemic report frequent concerns regarding their own health. Based on our knowledge, little information is however available regarding the impact on HCPs awaiting a test result for potential COVID-19 infection or interventions for supporting them during this waiting time. Therefore, this study aim to shed light on HCPs’ experiences of awaiting a test result for a potential COVID-19 infection through individual interviews. This qualitative investigation will thus highlight what is at stake for HCPs while in quarantine and awaiting a response as to whether they are infected with the coronavirus. The study offers an in-depth understanding of the meaning of the waiting for the test result for COVID-19 infection from the HCPs’ perspective and should be of interest to a broad readership and add knowledge to the growing COVID-19 evidence base and in developing supportive inetrventions targeted HCPs in such a pandemic.

While HCPs, e.g. nurses, physisians, porters and healthcare workers, are caring for some of the most vulnerable groups of people both in hospital but also in primary care, they are currently also facing an unprecedented disease caused by the outbreak of a previously unknown virus [6]. This new coronavirus that can cause COVID-19 disease [7] puts HCPs in a position where they must avoid exposing themselves to infection but also avoid transmitting the infection to the vulnerable patients and citizens to whom they have a caring responsibility. Because an infected HCP is a potential vehicle for virus dissemination, research suggests that reducing the risk of infection amongst HCPs is essential [8]. Spread of virus has been reported during the Ebola outbreak resulting in a compromised healthcare system [9] as well as during the Severe Acute Respiratory Syndrome (SARS) [10] and the Middle East Respiratory Syndrome (MERS) epidemics [11]. Experiences from these previous outbreaks highlight fear among HCPs in transmitting the disease and the importance of screening for the virus.

On 30th January 2020, the World Health Organization declared the Chinese outbreak of COVID-19 to be a Public Health Emergency of International Concern. The emergency committee stated that the spread of COVID-19 may, among other preventive efforts, be interrupted by early detection and isolation [7, 12]. General hygiene precautions are crucial in order to minimize the risk of contamination [8]. HCPs have always played an important role in infection prevention, infection control, isolation, containment and public health, which for nurses initially was advocated for by Florence Nightingale [13].

There are studies that define the pathophysiological characteristics of COVID-19 however, the mechanism of spread is uncertain. Current knowledge is derived from similar coronaviruses, which are transmitted from human-to-human through respiratory infection [7]. Typically, respiratory viruses are most contagious when a patient is symptomatic. However, increasing evidence suggests that human-to-human transmission may be occurring during the asymptomatic incubation period of COVID-19 [14, 15]. The disease is reported to be very contagious, and measures to reduce person-to-person transmission of COVID-19 are therefore required to control the outbreak [14–16]. Special attention and efforts to prevent or reduce transmission is applied in susceptible populations including HCPs in order to reduce transmission to patients or other vulnerable groups of people in the community [17–19].

HCPs are thus among those groups of people who are being rapidly tested for coronavirus in Denmark. Considering the severity of infection and illness [20], the test result might be of great importance for the healthcare system but also for the individual HCP. A sudden decrease in the number of HCPs because of quarantining or isolation due to COVID-19 infection would potentially overload the healthcare system and the capacity to treat either patients with coronavirus or patients with other serious conditions would be challenged [8]. For the individual HCP, it might furthermore be a threat to their own health. As far as we are aware, no research has so far focused on how HCPs might perceive this test situation. Therefore, the purpose of this study is to explore HCPs’ experiences of awaiting a test result for a potential COVID-19 infection. Such knowledge from the HCPs’ perspective are expected to increase the awareness of potential needed support while awaiting a crucial test result from a contagious and rare virus. Furthermore, the study will help hospital managers to establish strategies to ensure the best possible working conditions for HCPs during the pandemic.

## Methods

This study used a phenomenological hermeneutical methodology inspired by Ricoeur’s narrative philosophy [21]. In this study phenomenology was apllied as an epistemological stance for exploring first-person accounts of what it is like to wait for a test result for potential COVID-19 infection. Pre-reflexive experiences from the participant’s lifeworld is the starting point, while hermeneutics was focused on interpreting the surplus meaning contained in this lifeworld. As human beings we leave traces when we express ourselves, and these traces are formed by the meanings and traditions to which we belong. Often, it is impossible to directly understand individual’s experiences because the sense in the traces is hidden. Therefore, reflection on an individual’s lived experiences takes place via the narratives expressed by the individuals [21, 22]. The threefold mimesis is central in Ricoeur’s narrative philosophy and can be seen as an epistemological approach for understanding the participants’ lived experiences [23], which, in this study, has inspired the research process as a three-fold process [22]: Mimesis I (prefiguration): the life lived before it is formulated as spoken or written narrative (data collection); Mimesis II (configuration): the language stage, formulating a narrative (from speech to text); and Mimesis III (refiguration): the comprehension stage, when the text is interpreted (analysis and interpretation) [21–23].

### Sample

Participants in this study were recruited from a population of HCPs who had been tested for coronavirus but who did not necessarily care for COVID-19 patients. If they had symptoms of COVID-19 infection, HCPs in Denmark were offered testing for the virus. We used a convenience sampling strategy [24] by encouraging tested HCPs to approach the research team by e-mail if they were willing to attend an interview. The interviews were conducted by telephone based on ethical accountability for not contributing to the spread of the virus and they were scheduled in the gap between test and its result. The result of the test was during the study period given to a tested person within 24 h. The society of Denmark was on lockdown due to the threat of coronavirus on March 11th 2020. Coronavirus was in this period still relatively new in Denmark, and 300–500 patients were hospitalized and 77 patients died due to COVID-19 during week three of the epidemic. Fifteen HCPs agreed to participate in the study and were interviewed in March and April 2020. Thereafter data saturation was achieved, making further interviewing unnecessary [24]. We included HCPs with different professional backgrounds and different responsibilities from both primary care and hospitals. The characteristics of the participants are shown in Table 1.

**Table 1:** Characteristics of participants

Participants	*N* = 15
Sex:	
Female	11
Male	4
Age (years) meadian (range):	45 (20–64)
Profession:	
Nurse	8
Physician	1
Porter	2
Healthcare worker	4
Employment setting:	
Hospital	7
Primary care	8
Corona test result:	
Positive	3
Negative	12

### Data collection

Data were collected through individual interviews. Human events are characterized by unreflecting preunderstanding, which Ricoeur calls prefiguration (Mimesis 1) [21, 22]. With the aim of gathering the participants’ in-depth narrative accounts of their experiences of awaiting a COVID-19 test results, open questions were used. Each interview began with a broad opening question, such as; “Could you please tell me what led you to being tested for a potential COVID-19 infection and your experiences while awaiting the test result?” Table 2 lists the interview questions. The interviews lastet on average 30 min (range 9–55 min).

**Table 2:** Interview questions

Could you please tell me what led you to being tested for a potential COVID-19 infection?	
Could you please tell me about your experiences from awaiting the test result?	
Have you been quarantined / isolated, and if so, can you tell what it’s been like?	
Have you been in contact with healthcare professionals, and if so, what are your experiences about receiving health care?	
How do you feel about the risk of being infected?	
Are you thinking about whether you may have infected others?	
How do your surroundings respond to the possibility of you being infected?	
Do you know any others who are infected, and if so, what are your experiences with that?	

The interviews were separately conducted by three experienced qualitative researchers who all had a professional background as registret nurses, and interviews were audio-recorded and transcribed into 217 pages. The participants’ stories were thus transcribed into a textual configuration of their unarticulated experiences (from prefiguration to configuration) [21, 22]. According to Ricoeur, people’s narratives contain surplus meaning and hermeneutics is concerned with interpreting this surplus meaning (from configuration to refiguration).

### Ethical considerations

The study was undertaken in accordance with the guidelines of the Danish Ethical Research Committee and was approved by the Danish Data Protection Agency (P-2020-276). The investigation conforms with the principles outlined in the Declaration of Helsinki [25]. The participants received written information about the purpose of the study and their right to withdraw at any time. Written informed consent was obtained from each of the participants before the interview. Data were anonymized by means of identification codes. The participants were informed that interview data would be treated confidentially.

### Analysis and interpretation

**Fig. 1:**
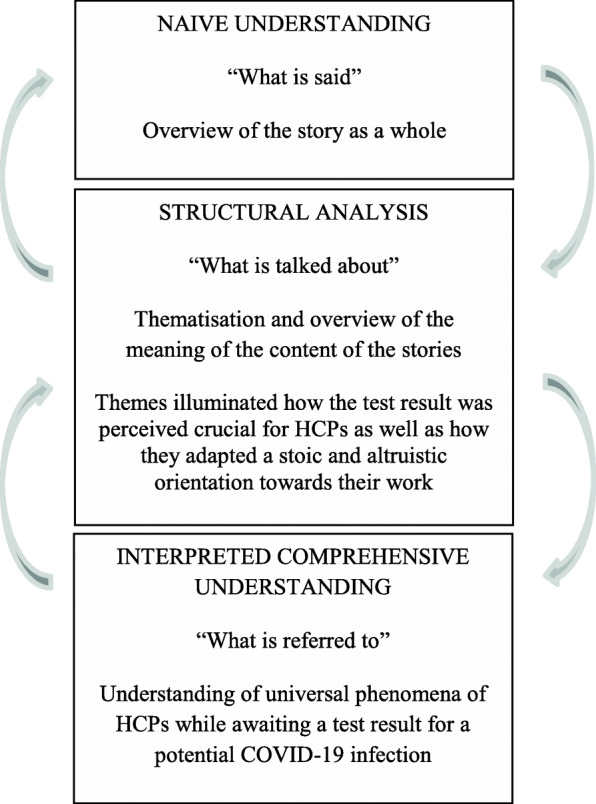
Illustration of the analysis and interpretation process

According to Ricoeur, interpretation is the central methodology in phenomenological research. Interpretation involves a process consisting of naive interpretation, structural analysis, and comprehensive understanding [26]. *Naive interpretation* is superficial interpretation, whereby the narratives are read and re-read to see what the texts mean to the researchers, giving an overall view of the narratives. *Structural analysis* deals with patterns in the text that can explain what it is saying. Explaining what the text expresses means moving from what the text says to what it is talking about. During the structural process, we analyzed and structured the narratives based on units of meaning, extracting meaning or themes that recurred in the narratives. The units of meaning were condensed such that the essential meaning was expressed. These units of meaning were then further condensed and gathered into themes [22, 26]. The *comprehensive understanding* continues with a discussion of the themes that were identified in the structural analysis, the purpose being to reach a new understanding of the possible dimensions of the participants’ experiences while awaiting a COVID-19 test result. The deeper interpretation of the narratives is a process of understanding in which theoretical perspectives are drawn on to help clarify and comprehend phenomena in the participants’ experiences [22, 26]. See Fig. 1.

### Rigour

Throughout the study methodological rigor was attained by using the qualitative concepts of relevance, validity, and reflexivity, as described by Malterud [27]. This study is one of only a few qualitative studies exploring the lived experiences of HCPs during the COVID-19 pandemic and to our knowledge this is the first qualitative study exploring HCPs’ experiences of awaiting a test result for a potential COVID-19 infection. The qualitative interview method was selected in order to gain insight into these individuals’ perspectives in order to understand the meaning of the investigated phenomena, i.e. the transition from experience to meaning [26]. The relevance of the study and the chosen methodology thus seems appropriate. Several strategies were employed to demonstrate internal validity, including collecting in-depth data, prolonged involvement with the data and use of the participants’ own words to formulate and illustrate themes. The participants are quoted in order to ensure transparency and substantiate the findings of the study. Ricoeur’s steps in the analytical process are clearly set out and have been stringently followed. The process from prefiguration through configuration to refiguration reflects the shift from lived life to narrative accounts of lived life to the final interpretation, which provides an insight into the individual HCPs’ concrete experiences and into universal phenomena of life for HCPs awaiting a test result. Thus other researchers are able to judge and validate the extracted themes. Reflexivity was ensured by discussions between the authors, both during the data collection phase and in the analysis. The fact that all interviewers were registered nurses meant that a certain agreement but also equality between participant and interviewer was present. This meant that the conversation was relatively easy and straightforward. In order, however, to prevent blind spots in relation to the research purpose, the interviewers were particularly aware of their role as researchers and qualitative interviewers and tried to bridle preunderstandings from their background as HCPs and adapting a curious stance.

## Results

The comprehensive understanding illuminated the meaning of the participants’ experiences of awaiting a COVID-19 test result as a stoic and altruistic orientation towards their work. These HCPs presented a strong professional identity overriding most concerns about their own health. The result of the coronavirus test was a decisive parameter for whether healthcare professionals could return to work. Experiences related to the test situation as well as the strong sense of professional identity will be described in more detail in the following.

### The crucial test response

What led the participants to the test for coronavirus were their experiences of mild to moderate symptoms, which aroused suspicion of possible infection. They described the importance of protecting patients, vulnerable citizens and colleagues from the risk of infection and therefore stayed away from work until they were certain that they were not contributing to the spread of the virus. This distance from work, however, had an impact on participants who described a dilemma in terms of both feeling responsible and hypochondriac at the same time. As HCPs they already knew the usual workload and therefore described feelings of failing colleagues by not taking part in the work, *“We are busy in healthcare, so if there is one who is sick, then the others just have to run faster” (participant K)*. Thus, the test result was extremely important in terms of whether one could return to work and help one’s colleagues.

The participants, furthermore, talked of particular responsibilities in being prepared to care for and treat patients with COVID-19. They watched what is going on in the rest of the world in other healthcare settings where the epidemic of COVID-19 exceeded the healthcare systems’ resources. They were very concerned about their colleagues in other countries but at the same time had an altruistic view that they themselves must also be prepared. In this context, coronavirus tests are also particularly important for the participating HCPs. They did, however, describe an ambivalence around the test response; if you are tested positive, then hopefully you will form some kind of immunity and thus be able to go to work after a period of quarantine without being infected again. If, on the other hand, you are tested negative, you can return to your job immediately, *“I hope I don’t have corona, but on the other hand, then you have had it …*” *(participant C)*. Participants describe concerns and fears that many HCPs will be infected at the same time, and that there will be no one to take care of the ill patients or vulnerable citizens. Therefore, it was necessary to have the HCPs tested so that an overview of the workforce can be maintained as HCPs cannot easily be replaced. The way to being tested could, however, be quite obscure for some of the participants. For participating HCPs working in the hospital, access to testing is easy and straightforward. They noticed symptoms, they discussed it with their boss, and they got tested. However, working in primary care posed major problems in figuring out access to being tested. Those HCPs narrated experiences of not being taken seriously, which produced a kind of powerlessness, *“All of us who work in healthcare, we are there to make a difference, but you just feel that we sometimes are banging our head against the wall [experiencing lack of understanding] … It gives a sense of powerlessness” (participant E)*. They furthermore described frustrations of wasting precious time waiting to get to the test; time that could have been spent usefully in continuing their work. The particular commitment to caring for vulnerable and ill people was evident when participating HCPs were just waiting to be tested.

Even though being tested for coronavirus when experiencing symptoms was strongly preferred by the participants in this study, the test situation, however, reminded and confronted them with the seriousness of the pandemic. They described their experiences of coming into the interimistic tents outside the hospital and meeting with test staff in protective equipment. The participants, being HCPs, were prepared for this scenario but are anyway confronted with feelings of being part of a surreal experience or a science fiction movie but also that this new virus was real, *“It is a peculiar experience to meet another person who is covered from head to toe. You suddenly feel very dangerous” (participant F)*. They also, however, told of a professional set-up and that being tested provided certainty, tranquility and direction.

### A stoic and altruistic orientation towards work

The participating HCPs in this study presented a strong sense of professional identity and were highly oriented towards their work. They talked about how they were preparing for battle against the coronavirus despite the risk of being infected themselves. The frontline HCPs with the critical task of caring for COVID-19 patients told how for a long time and with no evidence of even having the disease, they had isolated themselves at home, *“I already decided 14 days ago that we should stop sleeping in the same room and avoid physical contact completely. I have also written on my wife’s and my behalf to family and friends that we will not be able to see anybody for a while”* (*participant B*). They were tremendously aware of their specific role and duty and that nobody could stand-in for them and explained it as just being a part of their job and with a fatalistic attitude. These participants expressed a paramount need to know if they were contagious.

Common to the participants was that, by virtue of their profession, they had important professional knowledge about drop infections, hygiene, symptoms and pathways of infection, all of which gave them a readiness to act. They narrated how they were extremely aware of not transmitting the infection to others, as well as how to take distance and hygiene measures when they noticed symptoms of potential COVID-19. These measures seemed to be integrated as an almost natural act in the participants’ lives with them not questioning the necessity of doing so, *“I’ve locked myself inside a room now and told the others in the family to stay away. And if I’m going to the toilet ..., our apartment is quite small ... but then I just shout that now I go to the toilet. And then I have hand sanitizer and cleansers and wipe it all off afterwards” (participant C)*. The situation thus appears to have been tackled with stoic calm by the participants as they awaited answers as to whether their possible symptoms are related to COVID-19. Despite their professional knowledge, participants also told of chaotic and conflicting information from the healthcare system expressed as an information flow that had become incomprehensible and overwhelming. This resulted in uncertainty and difficulty in keeping up with guidelines.

The participants’ social network was marked by the possible threat of COVID-19 from the HCPs who were just doing their job in healthcare. The participating HCPs were highly aware that their family and friends were having a hard time knowing that the COVID-19 infection risk was a necessary condition of their job, while they at the same time are forced to keep a distance. This concern did not, however, cause participants to falter in their belief that they were doing the right thing by focusing on their core area, which was caring for ill and vulnerable people. The threat to their own health ran though the minds of the participants once in a while, *“That people who take care of their work and do what they can to make others survive can end up getting infected with COVID-19 themselves, I think that’s a little hard, but that’s just how it is” (participant G)*. The participating HCPs express a need to share such thoughts with somebody and ask for some kind of follow-up or a HCP corona hotline, e.g. after being tested for the virus, *“When you are nervous and scared, it would be helpful if you could go to one specific place where knowledge and expertise about corona was gathered - a mental health corona hotline” (participant D)*.

Being oriented towards their job was described as a natural part of the participating HCPs approach to life. They had a strong passion for and pride in their work and in this epidemic context showed solidarity across professional boundaries. They did question if they may be too uncritical but explained it with the fact that they are in a time when it is necessary to do as one is told. The participants, however, described how they have experienced the community tribute e.g. public applause for them as on the edge of hypocrisy. They rejected more applause from society and express how genuine societal recognition would be more resources in hospitals to solve problems and to give the HCPs a tolerable everyday life and a decent salary.

## Discussion

Awaiting a COVID-19 test result for the participating HCPs was associated with a stoic and altruistic orientation towards their work in which the result of the test was crucial. This study illuminated how HCP prepare and get ready for battle against COVID-19 in a devoted and solidarity-based way. This war metaphor as a response to the pandemic might illuminate the HCPs’ stoic and altruistic work identity. Seeing the coronavirus as an enemy that should be defeated and as a part of one’s job require HCPs who approach their work with a stoic calm and an altruistic attitude. A similar commitment to supporting their health system and communities has been reported during the Ebola epidemic [28]. The participants in our study presented a strong professional identity and their attention was directed to caring and protecting patients and vulnerable citizens while also preventing the spread of infection among colleagues. Being stoic in their approach to work does not mean that HCP are cold and distant, it is rather an attitude of remaining calm and carrying on and may also involve having a certain degree of self-control and maintaining a sense of conscious self-awareness [29]. The altruistic attitude or behavior of the participants was characterized by the fact that the individual sought to promote the well-being of others without thought for their own interests and needs. According to Hume, altruism is a character trait of humans that normally extends to strangers only in a weakened form and it is rare to meet with one in whom the affections of altruism do not over-balance the selfish [30]. Altruism was, however, a strong moral part of the participants’ professional identity which seems to be based on the inner logic of the HCP discipline. Understanding of the roles altruism might play in the social and medical response to an epidemic and the stories about the nature of HCPs’ moral obligations has been discussed and implies the willingness to take personal risks in the line of duty [31].

A professional identity can be defined as a social identity that relates to people’s understanding and presentation of themselves as professionals [32]. It is seen as the identity a person has developed through learning and practicing a given profession and thus can fulfill a particular employment function designed and integrated into a given work and professional culture. According to Goffman, identities are not created individually, but rather the individual gains his or her professional identity through the attribution of certain characteristics that have the character of normative expectations [33]. In addition to performing the expected functions associated with a specific field, the individual thus supports and supplements his or her position by simultaneously playing the normatively expected role associated with that group [33].

To follow Goffman [33], the stoic and altruistic orientation towards their work presented by HCP in the present study might also point to these HCP acting in accordance with a specific role within a given social context, such as healthcare. Society’s normative expectations of HCP may influence their perception of their own professional identity. Our study, however, illuminates a discrepancy between an altruistic role as HCPs and the normative expectations that come from the community that pays tribute to them, and then an experience of working conditions and salaries that do not indicate recognition. Altruism has been reported to be declining in the face of economic and pragmatic motivation [34] which might threaten healthcare practice during an epidemic such as COVID-19. Another threat to our study participants’ stoic and altruistic orientation towards their work was also experiences of receiving chaotic, conflicting and an overwhelming information flow resulting in difficulties in keeping up with best practice guidelines. Research from the A/H1N1 influenza pandemic have demonstrated how perceived sufficiency of information was associated with reduced degree of worry and how HCPs less frequently felt unprotected [35, 36]. These points highlight that hospital managers should try to provide and direct information for HCPs according to what is needed during the different and specific phases of a pandemic based on the affected HCPs’ perspectives in order to offer favourable working conditions in times of extreme distress.

Being tested for coronavirus for the HCPs in our study was significant in order to maintain their professional identity and continue working. They did, however, also describe experiences of uncertainty and fear for own health and expressed a need to share such thoughts with somebody. A threat to the mental health of HCPs during epidemics has been reported [4, 5, 37], and interventions to promote mental well-being in HCPs exposed to COVID-19 are suggested to be immediately implemented [37]. A hotline for patients during the current COVID-19 outbreak has been established in some places, e.g. in New York where citizens are guided to assess their own symptoms at home and can discuss any psychological impact from the disease [38]. Similar initiatives directed at HCPs are needed. Recomandations from a recent systematic review also suggest to establish a forum for medical personnel to voice their concerns as well as a psychological assistance hotline comprised of volunteers who have received relevant psychological training to be able to provide telephonic guidance to personnel to help effectively tackle mental health problems [39].

### Strengths and limitations

Telephone interviews in this study were unavoidable due to the risk of virus transmission between participants and interviewers. Such interviews do, however, have some disadvantages. They are more impersonal in that it is not possible to have eye contact, and as an interviewer, it is difficult to show that you are interested and included in what is being said. In addition, breaks are generally less acceptable [24]. Despite this, we found that participants were willing to participate in the study and appreciated talking about their experiences. The sample included in this study consisted of more female HCPs (*n* = 11) and most were nurses (*n* = 8) which might be an uneven distribution of participants. Women, however dominate the nursing profession, and nurses are the largest professional group in healthcare [40, 41] and the sample thus represents the general healthcare workforce.

What is worth noting is that this study was conducted during the first phase of the pandemic. This means that the stoic and altruistic orientation as well as the war metaphor that we have found and described may change over time as the pandemic progresses and HPCs may experience burnout.

## Conclusions

The perspectives of HCPs awaiting a test result for coronavirus provide an important contribution to the growing body of literature about COVID-19. These HCPs had a strong professional identity with their attention directed towards caring and protecting patients and vulnerable citizens while also preventing the spread of infection among colleagues. A discrepancy between an altruistic role as a HCP and the normative expectations that come from the community was also illuminated. The clinical implications of this study is thus, that as a stoic and altruistic attitude dominated HCPs’ identity, access to testing for COVID-19 for these professionals is crucial. Furthermore, a mental health corona hotline for HCPs should be established.

## Data Availability

All authors have full control of all primary raw data (interview transcripts) and allow the journal to review our data if requested. All raw data are written in Danish. Data are stored in a locked file cabinet in a locked room at the Copenhagen University Hospital as requested by the Danish Data Protection Agency. The data material used in this study are available from the corresponding author on reasonale request which will not conflict with the anonymity and confidentiality of the data.

## Abbreviations

HCP: Healthcare professionals
